# Exploring the protective effects of herbal monomers against diabetic retinopathy based on the regulation of autophagy and apoptosis: A review

**DOI:** 10.1097/MD.0000000000035541

**Published:** 2023-10-27

**Authors:** Zhuoyu Hu, Xuan Wang, Qi Hu, Xiangdong Chen

**Affiliations:** a Department of ophthalmology, The First Hospital of Hunan University of Chinese Medicine, Changsha, People’s Republic of China; b Graduate School of Hunan University of Chinese Medicine, Changsha, Changsha, People’s Republic of China.

**Keywords:** apoptosis, autophagy, diabetes retinopathy, traditional Chinese medicine monomer

## Abstract

Diabetic retinopathy (DR) has become one of the top 3 blinding eye diseases in the world. In spite of recent therapeutic breakthroughs, it is not yet possible to cure DR through pharmacotherapy. Cell death is thought to play a key role in the pathogenesis of DR. Moderate modulation of cellular autophagy and inhibition of apoptosis have been identified as effective targets for the treatment of DR. Numerous phytochemicals have emerged as potential new drugs for the treatment of DR. We collected basic DR research on herbal monomers through keywords such as autophagy and apoptosis, and conducted a systematic search for relevant research articles published in the PubMed database. This review provides the effects and reports of herbal monomers on various DR cellular and animal models in vivo and in vitro in the available literature, and emphasizes the importance of cellular autophagy and apoptosis as current DR therapeutic targets. Based on our review, we believe that herbal monomers that modulate autophagy and inhibit apoptosis may be potentially effective candidates for the development of new drugs in the treatment of DR. It provides a strategy for further development and application of herbal medicines for DR treatment.

## 1. Introduction

Diabetic retinopathy (DR) is a major complication of diabetes and is characterized by hard exudates, neovascularization, and vitreous hemorrhage.^[1]^ The incidence of diabetic macular edema and/or proliferative DR may lead to blindness in diabetic patients.^[2]^ It is estimated that 700 million people worldwide will suffer from diabetes in 2045, and 35% of diabetes patients will suffer from diabetes retinopathy.^[3,4]^ DR also leads to life-threatening vascular complications in the organism.^[5]^ The pathophysiology of DR is still not completely understood.^[6]^ However, the emerging evidence has pointed out a correlation between oxidative stress and other major metabolic abnormalities associated with DR development.^[7,8]^ Furthermore, the relationship between autophagy and apoptosis determines the extent of cellular oxidative stress and the progression of DR.^[9–11]^

The quality of life of patients with DR is significantly affected, but current pharmacologic treatment regimens are based on retinal laser coagulation, hypoglycemic agents, vascular endothelial growth factor (VEGF) therapy, and surgery.^[12]^ Consequently, how to target the prevention and control of DR and protect the visual function of patients has become one of the important tasks of modern medical research.

The main pathological features of DR are dysfunction of microvascular cells, apoptosis, and protein secretion imbalances in the extracellular matrix.^[13]^ In a high-glucose and hypoxic environment, oxidative stress generated by mitochondria can damage retinal nerve cells and vascular endothelial cells, especially retinal ganglion cells (RGC).^[14]^ Mitochondrial autophagy is a selective autophagy, a process by which cells selectively remove mitochondria through autophagy. Excessive mitochondrial autophagy and defective mitochondrial biosynthesis can lead to reduced mitochondrial content and ultimately cell death.^[15]^ It has been shown that cell death is closely associated with the development of DR.^[16]^ In this case, cell death mainly refers to regulatory cell death. Regulatory cell death, which is a regulatory response to external stimulation. It is a mode of cell death involving many proteins and induced by signal cascade, such as apoptosis, autophagy, etc.

Since cell death has been reported to be closely associated with the progression of DR, we have searched for new medicinal plants with therapeutic properties for the treatment of DR. Several herbs have been studied in randomized controlled clinical trials with animal studies, so we highlight among them resveratrol (RSV),^[17]^ sodium aescinate^[18]^ and astragalus polysaccharide.^[19]^ There is a rising interest in Chinese medicine for treating DR because of its multi-component, multi-target, multi-pathway efficacy and stable efficacy. Considering the relationship between herbal medicines and the pathophysiology of DR, we present in this work the relationship between autophagy, apoptosis and diabetes retinopathy. We also discuss the monomers of traditional Chinese medicines (TCM) that have the ability to regulate autophagy and inhibit apoptosis in DR. In summary, the purpose of this study is to provide theoretical basis for the prevention and treatment of diabetes retinopathy by TCM (Table 1).

**Table 1:** Chinese medicine monomers for the treatment of diabetic retinopathy.

Chinese medicine monomers	Mechanism	In vivo	In vitro	Autophagy or apoptosis
Artemisinin dericative	Beclin-1↑, LC3II/I↑, p62↓, AMPK↑, SIRT1↑	SD Rats	NA	Autophagy
Resveratrol	ROS↓p-AMPK↑, Sirt1↑, PGC-1α↑, Caspase-3↓	NA	Bovine retinal capillary endothelial cells (BRECs)	Apoptosis
Epigallocatechin-3-gallate	Beclin-1↑LC3-II↑P62↓caspase 3↓mTOR↓	SD rats	Müller cells	Autophagy
Gingerol	VEGF↓, NF-κB↓, iNOS↓,BA↓,caspase-3↓,Bcl-2↑,eNOS↑,G6PDH↑,GR↑,GSH↑,GPx↑,SOD↑,CAT↑,MDA↑,TNF-α↑,NF-κB↑.	Wistar rats	NA	Apoptosis
Ferulic acid	P53↓,BAX↓,Bcl2↑	db/db mouse	ARPE-19 cells	Apoptosis
Naringenin	Ki67↑,PCNA↑,ROS↓,Bcl2↑,Bax↓,caspase3↓,GTPCH1↑,eNOS↑,BH4↑,TNFα↓,IL 1β↓,IL6↓	NA	Human (H)RECs	Apoptosis
Hydroxy saffron yellow A	IL-1↓,TNF-α↓,MDA↓,SOD↑,Nrf2↑,HO-1↑,Bcl-2↑,P53↓	Wistar rats	NA	Apoptosis
Gypenoside XVII	CAT↑,MDA↓, GSH-px↑,SOD↑,ATG5↑, Bcl-2↑, Beclin1↑, LC3I/LC3II↑, caspase-9↓,Bax↓,caspase-3↓,P62↓	db/db mice	NA	Autophagy and apoptosis
Arjunolic acid	SOD,↑ MDA↓, ROS↓, IL-1β↓,IL-6↓,TNF-α↓,caspase-3↓, Bcl-2↑, Bax↓, HO-1↑,AMPK↑, p-AMPK↑, mTOR↓, p-mTOR↓, LC3II/I↑, p62↓	SD rats	ARPE-19 cells	Apoptosis
Arbutin	TNF-α↓,IL-1β↓,IL-6↓,NF-κBp65↑,COX-2↑,Bcl2↑,BAX↓,caspase-3↓,PARP↓,LC3II/I↑,beclin1↑,SIRT1↑	NA	ARPE-19cells	Autophagy and apoptosis
Diosgenin	Bcl-2↑,Bax↓,caspase 3↓,TNF-α↓,IL-6↓,IL-1β↓,COX-2↓, p65↓,MDA ↓,SOD↓,GSH-Px↓,p-AMPK/AMPK↑,Nrf2↑,HO-1↑	NA	ARPE-19cells	Apoptosis
Berberine	GABA↑,PKC-α↑,Bcl-2↑,GABAAR↑	SD rats	NA	Apoptosis
Neferine	TAC↑,TOS↓,PCNA↓,VEGF↓	SD rats	NA	Apoptosis

Bcl-2 = B-cell lymphoma-2, BH4 = etrahydrobiopterin, eNOS = endothelial nitric oxide synthase, GABA = γ-aminobutyric acid, GTPCH1 = GTP cyclohydrolase 1, HO-1 = heme oxygenase 1, LC3 = light chain 3, MDA = malondialdehyde, mTOR = mechanistic target of rapamycin, NF-κB = nuclear factor kappa-B, PCNA = proliferating cell nuclear antigen, PKC-α = protein kinase C-α, ROS = reactive oxygen species, SIRT1 = recombinant sirtuin 1, SOD = superoxide dismutase, VEGF = vascular endothelial growth factor.

## 2. Methods

In this paper, we have reviewed the basic research on the treatment of herbal monomers on DR. Articles published in peer-reviewed scientific journals were included. Articles were excluded if they were not written in English or published in peer-reviewed scientific journals.

### 2.1. Search strategy

Literature searches were conducted from the build knowledge infrastructure database to December, 2022. We identified relevant studies in the literature by searching the PubMed database. The following keywords were used: DR, diabetic retinopathies in combination with each of the following terms: autophagy, apoptosis. Our review included the studies that analyzed both DR and herbal monomers, and both autophagy and apoptosis. Exclusion criteria included: Repeated publications; Meta-analysis; Systematic Review.

### 2.2. Study selection

We screened relevant articles and selected more articles to read based on title and abstract. We read articles concerning our topic and all included articles were carefully discussed in this review. In the initial search, there were 1565 articles, and 13 articles were ultimately selected and discussed after reading titles and abstracts.

### 2.3. Autophagy and diabetes retinopathy

**Figure 1.**
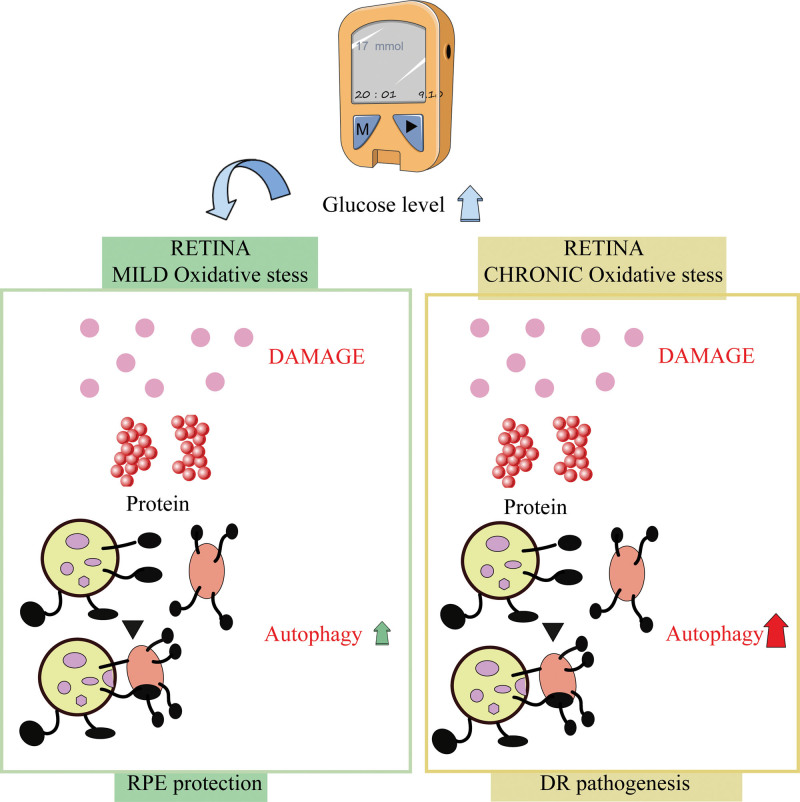
Scheme of the main autophagy changes related to diabetic retinopathy.

To maintain cellular homeostasis, autophagy removes misfolded or aggregated proteins and damaged organelles by removing them from the cell.^[20]^ Excessive autophagy or prolonged autophagy can lead to excessive destruction of proteins and organelles, disrupting the balance of cell metabolism and causing cell death^[21]^ (Fig. 1). As a result of high glucose levels, mitochondrial reactive oxygen species (ROS) are produced more readily and autophagosomes are formed, resulting in apoptosis and necrosis of endothelial cells in capillaries. Finally, it leads to intraretinal neovascularization, fibrosis and retinal detachment, etc.^[22]^ On the other hand, moderate autophagy is the body self-protection against stimuli, which can maintain the activity and number of islet B cells and enhance mitochondrial function, thus improving insulin resistance.^[23]^ When autophagy is impaired, Müller cells undergo apoptosis, which can be reversed by rapamycin.^[24]^ In retinal pigment epithelial cells, the mitochondrial deacetylase Sirt3 activates mitochondrial autophagy by Foxo3a/PINK1-Parkin pathway in a hyperglycemic environment.^[25]^ In general, autophagy is involved in the development of DR as a protective mechanism, closely related to ischemia and hypoxia, neovascularization, and insulin resistance.^[26,27]^

### 2.4. Apoptosis and diabetes retinopathy

According to recent studies, several apoptotic signaling pathways are involved in DR, diabetic peripheral neuropathy, diabetic nephropathy, and other diabetic complications.^[28–30]^ Apoptosis is an important factor in retinal microvascular disease and neuropathy in diabetic patients.^[31]^ At present, apoptosis occurs mainly via 3 pathways: mitochondrial, death receptor and endoplasmic reticulum pathways.^[32]^ Liu found that sarcoplasmic/endoplasmic reticulum calcium ATPase 2 is inactivated by irreversible oxidative modification of Cys674 residues, which disrupts calcium homeostasis in the mitochondrial pathway and promotes apoptosis.^[33]^

There are many factors that can cause endoplasmic reticulum stress, including hypoxia, calcium metabolism disorders, and oxidative stress, resulting in the accumulation of unfolded or misfolded proteins and ultimately apoptosis.^[34]^ In a DR model, high glucose causes retina damage via endoplasmic reticulum stress-induced apoptosis. Moreover, increased expression of endoplasmic reticulum stress-related C/EBP homologous protein and glucose regulated protein 78 was found in a related model^[35]^ Specifically, miR-30a inhibition led to both increased Fas expression in endothelial cells and increased migration of FasL-expressing microglia to ischemic injury sites to protected retinal neurons from ischemic injury.^[36]^ It was found that insulin-like growth factor binding protein-3 reduces tumor necrosis factor-α (TNF-α) levels by activating the c-Jun cascade, which helps to inhibit retinal microvascular endothelial cell apoptosis citation.^[37]^ We believe that TNF-α could be a new target for further investigation of DR-related anti-apoptotic drugs.

## 3. Results

### 3.1. Artemisinin dericative

Artesunate (ART) is a sesquiterpene lactone with a peroxybridge structure with antimalarial, antiviral, anti-inflammatory, antitumor and immunomodulatory pharmacological effects.^[38,39]^ ART can be used in the treatment of diabetes and diabetic complications, such as diabetic nephropathy, diabetic peripheral neuropathy and cardiovascular complications.^[40–42]^ In addition, ART reduced retinal thickness in diabetic rats significantly and attenuated retinal inflammation in diabetic rats through AMPK/Sirt1-mediated autophagy.^[43]^ Activation of adenosine 5’-monophosphate (AMP)-activated protein kinase (AMPK) can induce autophagy by directly suppressing the phosphorylation of the mechanistic target of rapamycin (mTOR).^[44]^ Recombinant sirtuin 1 (SIRT1) protects against diabetes-induced damage via suppressing inflammation and oxidative stress.^[45]^ Therefore, how to improve the bioavailability of ART and the efficacy of DR has become the focus of subsequent studies in this field.

### 3.2. Phenols

#### 3.2.1. Resveratrol.

In the terms of phytochemistry, RSV has anti-inflammatory, antioxidant, lipid regulating, and vasodilating properties.^[46–48]^ Li and coworkers have demonstrated that RSV inhibits endoplasmic reticulum stress, thereby significantly suppressing abnormal retinal vascular proliferation.^[49]^ According to Li, RSV inhibits apoptosis in bovine retinal capillary endothelial cells via AMPK/SIRT1/PGC-1 pathway to decrease reactive oxygen species, caspase-3, and reactive oxygen species.^[17]^ Apparently, herbal monomers are indispensable for alleviating retinal damage brought about by diabetes through cell death and cannot be separated from the regulation of the AMPK/SIRT1 pathway.

#### 3.2.2. Epigallocatechin-3-gallate.

In green tea, epigallocatechin-3-gallate (EGCG) is the main catechin.^[50]^ It can enhance autophagy, improve cellular clearance, and inhibit neovascularization.^[51]^ Müller cells cross the entire retinal layer, which makes them especially sensitive to damage in diabetes.^[52]^ Hence, Müller cell damage in diabetic patients contributes significantly to DR pathogenesis. Wang found that EGCG inhibited nuclear factor kappa-B (NF-κB) expression in high-glucose Müller cells after 3-methyladenine of an autophagy-specific inhibitor was added.^[53]^ It demonstrates that the passive effect of EGCG on p-NF-κB in diabetic rat retinal tissue and high-glucose cultured Müller cells was accomplished through activation of autophagy.

#### 3.2.3. Gingerol.

Ginger is one of the most popular spices in cookery preparations around the world, and the main active and pungent constituent of this plant is gingerol.^[54]^ In previous studies, ginger and ginger extracts containing 5% 6-gingerol have been shown to enhance retinal cell function by preventing oxidative damage and inflammation.^[55]^ Glucose-6-phosphate dehydrogenase deficiency is strongly associated with DR.^[56]^ Antioxidants and anti-inflammatory compounds have been reported to inhibit retinopathy and ameliorate the neurovascular injury normally associated with retinopathy.^[57]^ It has been reported that gingerol inhibited endothelial nitric oxide synthase (eNOS), G6PDH, oxidative damage, apoptosis, inflammation, and angiogenesis to improve DR.^[58]^ As a result, the use of antioxidant compounds and plant polyphenols such as ginger to control the inflammatory state and oxidative damage seems to be an adaptable therapeutic means.

#### 3.2.4. Ferulic acid.

Chinese herbs(e.g., ferulic acid, angelica, shengma, Ligusticum chuanxiong) contain ferulic acid (FA), a phenolic acid obtained from common plant species, which is the main monomeric active ingredient in a variety of water-soluble extracts.^[59]^ There is growing evidence that FA contributes to many serious diseases, such as cancer, Alzheimer disease, and diabetes-related vascular damage.^[60–62]^ Researchers found that FA protects adult retinal pigment epithelial cell line-19 cells and mouse retina from sodium iodate damage.^[63]^ Due to FA treatment, BCL2 associated X (Bax) levels were reduced and B-cell lymphoma-2 (Bcl-2) levels decreased in ARPE-19 cells induced by high glucose, as well as p53 activation was inhibited.^[64]^ In other words, FA inhibited HG-induced apoptosis of RPE cells through the P53/BAX/Bcl-2 pathway and ameliorated retinal tissue cell edema.

In summary, RSV, EGCG, gingerol and FA belong to the multiclassified plants. RSV is a stilbenol that is stilbene in which the phenyl groups are substituted at positions 3, 5, and 4’ by hydroxy groups.^[65,66]^ EGCG is a gallate ester obtained by the formal condensation of gallic acid with the (3R)-hydroxy group of (−)-epigallocatechin.^[67]^ It has a role as an antineoplastic agent, an antioxidant, a plant metabolite, a geroprotector and an apoptosis inducer.^[68]^ Gingerol is a beta-hydroxy ketone that is 5-hydroxydecan-3-one substituted by a 4-hydroxy-3-methoxyphenyl moiety at position 1.^[69]^ It has a role as an antineoplastic agent and a plant metabolite.^[70,71]^ FA is a ferulic acid consisting of trans-cinnamic acid bearing methoxy and hydroxy substituents at positions 3 and 4 respectively on the phenyl ring.^[72]^ It has a role as an antioxidant, a plant metabolite, an anti-inflammatory agent, an apoptosis inhibitor and a cardioprotective agent.^[73]^ It can be seen that antioxidant activity is one of the most widely utilized pharmacological effects of plant polyphenols. Natural polyphenolic compounds carrying phenolic hydroxyl groups have good antioxidant properties.^[74]^ So they have a strong scavenging capacity for free radicals (such as ROS).

### 3.3. Flavonoids

#### 3.3.1. Naringenin.

Naringenin (4’,5,7 trihydroxyflavone) is a native flavonoid compound and is distinguished by its high availability and low toxicity.^[75,76]^ Naringenin is effective in the treatment of obesity, diabetes mellitus, and corneal burns.^[77–79]^ The results of Xue study show that high glucose (HG) promotes human retinal endothelial cells (HREC) apoptosis, increases ROS production, and decreases the levels of the proteins GTP cyclohydrolase 1, eNOS, and etrahydrobiopterin on HRECs.^[80]^ As a result, an application of naringin upregulated GTP cyclohydrolase 1/eNOS signaling, stimulated etrahydrobiopterin release, and attenuated HREC injury significantly.

#### 3.3.2. Hydroxy saffron yellow A.

**Figure 2.**
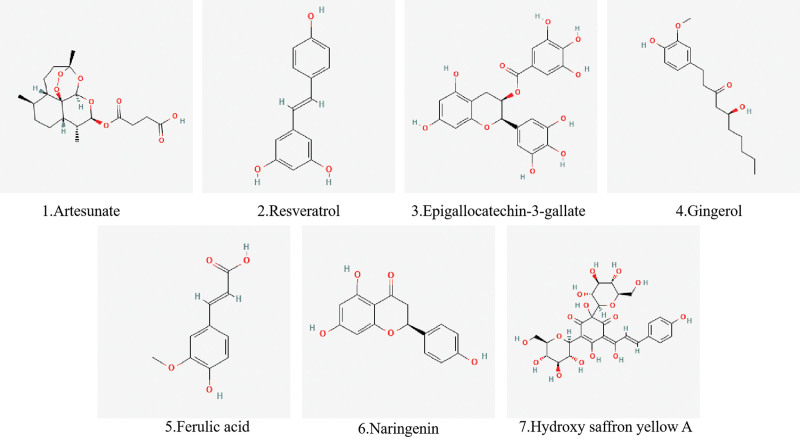
The chemical structures of artemisinin dericative, phenols, and flavonoids.

Safflowers are well-known herbs used to improve circulation, eliminate blood stasis, and treat cardiovascular and thrombotic diseases.^[81,82]^ Hydroxy saffron yellow A is the most significant bioactive component of saffron.^[83]^ Pathogenesis of DR is strongly linked to diseases such as vascular lesions, neuropathy, and inflammation, in which Nuclear Factor erythroid 2-Related Factor 2 (Nrf2)/heme oxygenase 1 (HO-1) signaling pathway-mediated inflammation and oxidative stress plays a key role.^[84]^ Sun found that through its activation of the Nrf2/HO-1 pathway, HYSA reduces retinal barrier damage caused by diabetes, inhibits apoptosis of RGCs, and reduces inflammation and oxidative stress damage.^[85]^ The chemical structures of Artemisinin dericative, Phenols, and Flavonoids are shown in Figure 2.

### 3.4. Triterpenoids

#### 3.4.1. Gypenoside XVII.

Gypenoside XVII (Gyp-17) is a saponin extracted from Panax ginseng (Burk) with few glycosyl groups and high adsorption capacity.^[86]^ In Müller cells, Gyp-17 treatment significantly decreased Bax, P62 and malondialdehyde expression and increased Bcl-2, autophagy protein 5, superoxide dismutase.^[87]^ Moreover, Gyp-17 primarily prevents inner nuclear layer damage and has no significant effect on other structural members of the retina. Consequently, Gyp-17 significantly decreases cell apoptosis, promoting autophagy, leading us to focus on Müller cells in the development of DR.^[88]^

#### 3.4.2. Arjunolic acid.

Colletotrichum cepa is a traditional Chinese herbal medicine, and arjunolic acid (AA) is a rich bioactive component of colletotrichum cepa with significant protective effects against various metabolic diseases including diabetic complications. The past studies have shown that AA may be an effective pharmacological agent for treating DR due to its anti-inflammatory, antioxidant, and anti-apoptotic properties.^[89,90]^ A dose-dependent effect of AA on ROS and malondialdehyde activity in DR rats was observed, as well as increased levels of superoxide dismutase and HO-1 protein expression.^[91]^ Meanwhile, AA significantly increased AMPK phosphorylation, upregulated light chain 3-II/light chain 3-I expression, and decreased mTOR phosphorylation.^[91]^ In a word, AA protected DR by activating the AMPK/mTOR/HO-1 autophagy pathway and inhibiting oxidative stress, inflammation and apoptosis.

### 3.5. Glycosides

#### 3.5.1. Arbutin.

Arbutin is an extracted component from the Chinese herbal medicine Arbutus bark, which is a naturally occurring soluble glycosylated phenol.^[92]^ It was demonstrated that Arbutin could protect cells from HG-induced injury and lessen H_2_O_2_-induced damage to optic ganglion cells.^[93]^ Ma found that Arbutin could protect adult retinal pigment epithelial cell line-19 from HG-induced damage, mainly by stimulating autophagy through SIRT1 and inhibiting HG-induced inflammation and apoptosis in ARPE cells.^[94]^ Therefore, based on the above mentioned herbal monomers, we identified SIRT1 as a potential target for DR treatment and confirmed that RSV, artemisinin derivatives, and arbutin induce autophagy in DR.

#### 3.5.2. Diosgenin.

Diosgenin is a steroidal saponin, obtained from the Chinese medicine yam, which is widely used in the treatment of cardiovascular, cerebrovascular, tumor, and diabetes.^[95–97]^ In addition to inhibiting mTOR, AMPK can also catalyze the phosphorylation of ser550 residues in Nrf2 protein, facilitating its nuclear translocation of Nrf2 and increasing the expression of downstream antioxidant proteins.^[98]^ It was also found that diosgenin elements attenuated HG-induced apoptosis, inflammatory damage and oxidative stress in ARPE-19 cells through activation of AMPK/ Nrf2/HO-1 pathway.^[99]^

Structurally, diosgenin is a sapogenin that is spirostan which is substituted by a hydroxy group at the 3beta position, contains a double bond at the 5 to 6 position, and has R- configuration at position 25.^[100]^ It has a role as an apoptosis inducer, an antiviral agent, an antineoplastic agent and a metabolite.^[95]^ Comparably, Gyp-17 is replaced by a hydroxyl group at position 3, a structure that may form a structure with inhibitory effects on apoptosis.^[101,102]^ Similarly, arjunolic acid is olean-12-en-28-oic acid substituted by hydroxy groups at positions 2, 3, and 23 (the 2alpha,3beta stereoisomer). So arjunolic acid and Gyp-17 have a role as a metabolite and an antioxidant.^[88,103,104]^

### 3.6. Alkaloids

#### 3.6.1. Berberine.

Berberine is a Rhizoma Coptidis extract, which has various effects such as anti-inflammatory, hypoglycemic, hypolipidemic and anti-tumor.^[105,106]^ While previous studies of DR have centered on pathological alterations in the retinal microvascular system, it has recently been suggested that diabetes may do harm to the neurons of the retina directly.^[107,108]^ Among all neurons, the RGC is most susceptible to damage, resulting in severe visual impairment.^[109]^ The early stages of DR were closely associated with increased and decreased neurotransmitters, as evidenced by an increment in glutamate release and a decline in γ-aminobutyric acid release.^[110]^ Fang found that Berberine potently defended against RGC apoptosis in the retina of rodent diabetic animals, and its defensive effect might be linked to the GABAARs/protein kinase C-α pathway activation.^[111]^ In conclusion, traditional Chinese medicine monomer can reduce the optic nerve injury of diabetes retinopathy by regulating apoptosis.

#### 3.6.2. Neferine.

**Figure 3.**
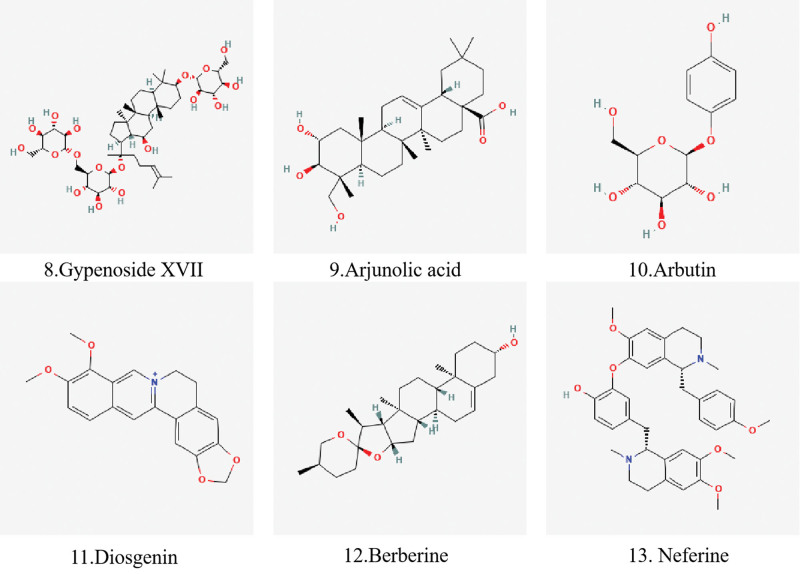
The chemical structures of triterpenoids, glycosides, and alkaloids.

Neferine is an alkaloid extracted from the seeds of the water lily family with anti-inflammatory, anti-diabetic and anti-tumor effects.^[112,113]^ Inhibition of high glucose-induced endothelial cell apoptosis by blocking the ROS/Akt/NF-κB pathway provides evidence for the use of Neferine in the treatment of diabetic microvascular injury.^[114]^ Furthermore, Neferine increased the expression of retinal total antioxidant capacity and down-regulated the immunoreactivity of retinal total oxidative stress, VEGF and proliferating cell nuclear antigen in diabetic rats.^[115]^ The chemical structures of triterpenoids, glycosides and alkaloids are shown in Figure 3.

## 4. Conclusion

In general, autophagy and apoptosis have been investigated as potential therapeutic targets for DR in numerous studies. In contrast to surgery alone or anti-VEGF therapy, herbal monomers for the treatment of DR have received extensive attention from academic researchers recently because of their mild side effects.^[116,117]^

**Figure 4.**
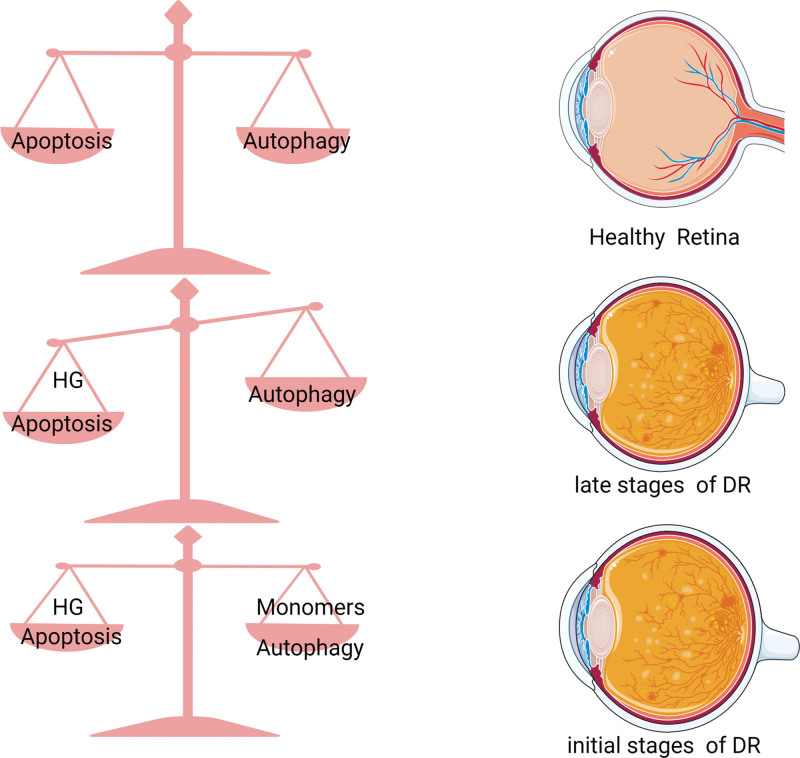
The dual role of autophagy in diabetic retinopathy.

Overall, autophagy has clearly become a research hotspot for DR.^[27,116]^ The majority of evidence indicates that autophagy plays a dual role in time and volume accumulation (Fig. 4). In mild stress or the early stages of DR, autophagy serves as an adaptable response with pro-survival and apoptosis-inhibitory effects.^[118]^ In contrast, during excessive stress and late stages of DR, the dysregulation of autophagy leads to apoptotic retinal cell death and exacerbates blood-retinal barrier damage due to the overloaded damage.^[119]^ Thus, autophagy and apoptosis are mutually regulated and in dynamic balance to maintain normal retinal cell physiology.

Chinese medicine is a cultural product that has come through thousands of years of Chinese medical theory and practice, and has had a profound impact on the Chinese people. A large number of biologically active compounds are an important basis for TCM. The development of active pharmaceutical ingredients in individual TCM is a fast way to develop a new class of drugs.^[120,121]^ In the era of big data, we list the herbal monomers related to DR in the form of literature management. We also present the prospects of exploiting secondary drugs and new applications of old drugs, as well as the key targets for developing new drugs with potential to treat DR. Consequently, we consider that the key to modernization of TCM is the development of drugs that can satisfy the clinical treatment needs of DR. This is a contemporary way that characterizes the development of highly effective new herbal monomers. The relevance of this for the prevention and treatment of DR can never be overemphasized.

## Acknowledgments

We would like to thank Dr Zou Menglong for polishing the language of the article.

## Author contributions

**Conceptualization:** Zhuoyu Hu, Xuan Wang, Qi Hu.

**Methodology:** Xuan Wang, Qi Hu.

**Resources:** Xiangdong Chen.

**Writing – original draft:** Zhuoyu Hu.

**Writing – review & editing:** Zhuoyu Hu.
